# Effect of Cognitive Control on Attentional Processing of Emotional Information Among Older Adults: Evidence From an Eye-Tracking Study

**DOI:** 10.3389/fnagi.2021.644379

**Published:** 2021-04-29

**Authors:** Haining Liu, Haihong Liu, Feng Li, Buxin Han, Cuili Wang

**Affiliations:** ^1^Psychology Department, Chengde Medical University, Chengde, China; ^2^Hebei Key Laboratory of Nerve Injury and Repair, Chengde Medical University, Chengde, China; ^3^Centre for Research in Psychology and Human Well Being Faculty of Social Sciences and Humanities, The National University of Malaysia, Bangi, Malaysia; ^4^School of Statistics, Jiangxi University of Finance and Economics, Nanchang, China; ^5^Research Center of Applied Statistics, Jiangxi University of Finance and Economics, Nanchang, China; ^6^CAS Key Laboratory of Mental Health, Institute of Psychology, Beijing, China; ^7^University of Chinese Academy of Sciences, Beijing, China; ^8^School of Nursing, Peking University, Beijing, China

**Keywords:** cognitive control, attention, emotion, positivity effect, eye-tracking

## Abstract

**Background:** Although numerous studies have suggested that the gradually increasing selective preference for positive information over negative information in older adults depends on cognitive control processes, few have reported the characteristics of different attention stages in the emotional processing of older individuals. The present study used a real-time eye-tracking technique to disentangle the attentional engagement and disengagement processes involved in age-related positivity effect (PE).

**Methods:** Eye movement data from a spatial-cueing task were obtained for 32 older and 32 younger healthy participants. The spatial-cueing task with varied cognitive loads appeared to be an effective way to explore the role of cognitive control during the attention engagement and disengagement stages of emotion processing.

**Results:** Compared with younger adults, older participants showed more positive gaze preferences when cognitive resources were sufficient for face processing at the attention engagement stage. However, the age-related PE was not observed at the attention disengagement stage because older adults had more difficulty disengaging from fearful faces than did the younger adults due to the consumption of attention by the explicit target judgment.

**Conclusion:** The present study highlights how cognitive control moderates positive gaze preferences at different attention processing stages. These findings may have far-reaching implications for understanding, preventing, and intervening in unsuccessful aging and, thus, in promoting active and healthy aging.

## Introduction

In the later years of life, various functioning areas in older adults, particularly in specific cognitive domains (e.g., cognitive control, speed of information processing, and memory), decline with age (Beaudreau and O’Hara, 2008). However, many older adults exhibit higher emotional stability rather than a weakening trend in cognitive functions; that is, emotional regulation ability increases with successful aging. One possible way to acquire this ability is by selectively deploying attention resources (Urry and Gross, 2010). The phenomenon that, compared with younger adults, older adults selectively attend to positive information rather than negative information in the environment is conceptualized as a “positivity effect” (PE) (Mather and Carstensen, 2005; English and Carstensen, 2015).

Socioemotional selectivity theory (SST), a noteworthy theory, maintains that goals direct social preferences (Mather and Knight, 2005). With the time horizon decreasing, older adults are motivated to commit to emotionally meaningful experiences and optimize positive affective feelings. SST on social, emotional, cognitive, and health-related processes has been continually and widely applied in new research areas to generate second-generation SST (Lang and Carstensen, 2002; Charles and Urban, 2015). The cognitive control hypothesis (CCH), an important component of second-generation theoretical models, specifically targets cognitive aspects and states that high-level cognitive control functioning is an essential prerequisite for older adults to successfully attain their emotional goals (Charles and Hong, 2017). To test the CCH, researchers have focused on variations in cognitive control in attentional emotional processing among older and younger individuals. The results have indicated that older adults with higher cognitive control performance tend to display PE (Knight et al., 2007). Once their cognitive resources are occupied by secondary tasks and become relatively scarce (i.e., when they have a working memory load) (Kennedy et al., 2020), their attention will no longer be drawn toward positive stimuli or away from negative stimuli. However, other studies have failed to demonstrate the CCH and found that during emotional picture categorization tasks, the stronger cognitive ability of older adults was associated with greater negative information processing in an oddball paradigm (Foster et al., 2013). Similarly, in eye-tracking studies, some scholars have argued that older adults’ cognitive control abilities predict the magnitude of their PE in the gaze (Allard and Isaacowitz, 2008), which is consistent with the CCH. However, others using pupil size as an indicator of cognitive effort have found that older adults make little effort to engage in a positive gaze when experiencing a negative mood (Allard et al., 2010). Their findings are seemingly confounded by the argument that PE is involved in top-down, voluntary cognitive control processes (Henderickx et al., 2009; Reed and Carstensen, 2012; Sakaki et al., 2019). One possible reason for these mixed findings is that age-by-valence PE is not only dependent on different task demands accounting for the cognitive load (Lavie, 2005; Mark and Murray, 2017) but also affected by different attentional engagement and disengagement processes (Demeyer et al., 2017).

Recent scholars have begun to focus on the interaction between cognitive control and different attentional processes in the age-related PE. However, further research is needed. First, empirical works have investigated this effect on attentional engagement and disengagement processes, whereas whether a PE occurs and in which stage remains unclear. Demeyer et al. (2017) used this task to assess the influence of different mood states on attentional processes. Their results showed that attentional disengagement processes (i.e., longer times in shifting attention away from negative stimuli) seem to be linked to age-related PE instead of attentional engagement processes. In addition, they proposed that this finding might reflect older adults’ intentional attentional deployment involved in the implementation of cognitive control in emotion regulation and emerging in later attention components—attention disengagement. However, in another study using the same engagement–disengagement task to measure attentional deployment processes, no evidence was found that different attentional processes are associated with the occurrence of age-related PE (Steenhaut et al., 2019). The engagement–disengagement task used in both of the above studies disentangles the two interrelated attentional components—attentional engagement and disengagement—across different conditions rather than in a timed sequence of attention processing. Second, previous studies evaluating cognitive load effects on the age-related PE have mainly focused on attentional disengagement from emotional faces (Demeyer et al., 2017; Steenhaut et al., 2019). Some findings have suggested that the different amounts of available attentional resources would account for the degree of covert shifts of spatial attention that are implicitly induced by the task (Lavie, 2005; Brassen et al., 2010). Specifically, Brassen et al. (2011) manipulated attentional load on emotional face distractors by explicitly varying covert endogenous orientation of attention through differentiating spatially valid, invalid, and uninformative cues in a modified spatial-cueing paradigm to examine the cognitive resource-dependent PE. The conflict in this paradigm is manipulated by the competition between the attentional preferences of older adults for happy faces and target-related stimuli (i.e., arrows with predictive target stimulus orientation) (Carretié, 2014). Cognitive control is considered to resolve conflicts through attentional bias in cognitive processing and amplifying task-related stimulus information (Egner and Hirsch, 2005). In addition, this task distinguishes high and low attention to emotional faces under two attentional conditions: the former refers to uninformative cues that allow relatively stronger face processing, whereas the latter refers to spatial cues that shift covert attention to the cued side of the image, resulting in attentional resources being reduced on emotional faces. Finally, in this paradigm, participants must first assign their attention resource to the cued location (e.g., where the happy, threatening, or neutral stimulus is presented), which involves attentional engagement processes, then shift attention away from the original position, and subsequently direct and reorient it to the target location, which involves disengagement processes of spatial attention (Posner et al., 1980; Brassen et al., 2010). Therefore, this paradigm is considered to be capable of separating attentional disengagement from attentional engagement in time sequences (Fox et al., 2002; Nummenmaa et al., 2006). Functional magnetic resonance imaging (fMRI) results showed that when more cognitive resources were attainable for facial processing, older adults were particularly easily distracted by happy faces. This effect was accompanied by enhanced activities of the rostral anterior cingulate cortex (Brassen et al., 2011). Although this study provided some neurobiological evidence that PE depends on cognitive control resources, it emphasized the importance of the cognitive control effect on attentional disengagement and did not differentiate the time processes of attentional engagement and disengagement in isolation. However, studies have emphasized the importance of the distinction between attentional engagement and attentional disengagement (Sanchez et al., 2013, 2014). Third, previous studies have stressed the importance of disentangling the internal associated components of attention, in particular attentional engagement and attentional disengagement (Brassen et al., 2010, 2011; Isaacowitz and Noh, 2011), but few have paid attention to the intermediate transition between two attention processes—covert attentional shift. For instance, the modified spatial-cueing paradigm used in a former study involved covert attentional shifting away from emotional faces (Brassen et al., 2011). One very influential viewpoint is that there is always an explicit spatial attention transfer after each covert shift in visual spatial attention (Perry and Zeki, 2000). Meanwhile, a short time delay in eye movement is followed by spatial attention shifts (Clark, 1999; Eimer et al., 2006), as it takes a certain amount of time to plan and perform eye movement to the gaze position, i.e., saccade latency. Given that eye movement is an explicit form of attention allocation (Chita-Tegmark, 2016), eye-tracking technology has become an invaluable tool to explore the function of the attention system from initial orientation and maintenance to subsequent disengagement from emotional faces without time delay (Nummenmaa et al., 2006; Kuhn et al., 2010).

Understanding eye movement characteristics in younger and older samples is important in expanding the findings of Brassen et al. (2011) and providing new insights into whether cognitive control differs in response to attentional engagement and disengagement processes. In the present study, we combined eye-tracking technology with a modified spatial-cueing paradigm (Brassen et al., 2011), which allowed us to manipulate the control levels of affective conflicts and thus cognitive resources for emotional distractors (i.e., facial expressions) indicated by an overt reflection of attentional deployment—eye movements (Armstrong and Olatunji, 2012; Chita-Tegmark, 2016). The aim of our research was to explore the influence of cognitive control on the different selective attentional processing stages of emotional information based on eye movement measurement to compare younger and older adult performance. Given the classic studies (Brassen et al., 2011), we hypothesize that when greater cognitive resources are available, healthy older adults, in contrast to younger adults, demonstrate a tendency toward preferential attention to positive faces, reflecting age-related PE. This study further explored different attentional stages of emotional information processing associated with PE by investigating eye movements recorded as an objective and quantitative index of attention allocation. Based on the CCH of second-generation SST, we speculate that at the attention engagement stage, older adults are more likely to be distracted by happy faces when more cognitive resources are available. In the attention disengagement stage, the PE of the older adults would not be observed, as their attention resources are occupied by covert endogenous cues.

## Methods

### Participants

The sample size was calculated using a feasibility web-based application named Power ANalysis for GEneral ANOVA designs (PANGEA) version 0.2^1^. In light of a previous study (Isaacowitz et al., 2009) that reported an effect size of Cohen’s *d* = 0.66 for the difference in positivity bias effect sizes between older and younger samples (Reed et al., 2014), we assumed the same effect size in the present population. Sixteen participants per group were determined at α = 0.05 and 1 - β = 0.80 based on the crucial parameters of vision during the attention disengagement stage (i.e., saccadic latency). Taking non-trackable participants into consideration, 42 community-dwelling older adults aged 61–84 years were recruited through telephone interviews, and 34 college-aged adults aged 18–24 years were recruited through online advertisements. Both groups were healthy, without current or past nervous system diseases or mental illness. All subjects reported being right-handed. The visual inspection results showed that their vision was normal or corrected to normal. All of their cognitive abilities reached normal values on the Chinese version of the Montreal Cognitive Assessment (MoCA) with reference to education-corrected norms. Ten of the 42 older participants failed to calibrate or tracked for ≤30% of the trials because of visual deficits (i.e., drooping eyelids and yellowed lenses), leaving 32 participants (76.19% of the sample) for the final analyses. The excluded older participants did not differ from the final older samples in terms of age [*t*(31) = 0.32, *p* > 0.05], sex (χ^2^ = 0.12, *p* > 0.05), years of education [*t*(31) = 1.08, *p* > 0.05], MoCA scores [*t*(31) = 1.74, *p* > 0.05], Positive and Negative Affect Schedule positive affect (PANAS-PA) subscale scores [*t*(31) = 0.36, *p* > 0.05], PANAS negative affect (PANAS_NA) subscale scores [*t*(31) = 0.96, *p* > 0.05], and Digit Span scores [*t*(31) = 0.63, *p* > 0.05]. Two of the 34 younger participants failed to calibrate due to hard contact lenses, leaving 32 younger participants (94.12% of the sample) for eye movement analyses.

### Measures

#### MoCA

The Beijing version (Lu et al., 2011) of the MoCA was modified based on philological and cultural changes from the original English version^2^. It includes multiple cognitive domains, such as visuospatial abilities, language, memory, attention, executive function, concentration, and orientation; totals 30 points; and is administered in 10 min. The MoCA Beijing version was an appropriate screening tool to detect mild cognitive impairment (MCI) in developed Chinese cities or other areas. It showed good criteria validity (Pearson correlation coefficient with Mini-Mental State Examination = 0.83) and acceptable internal consistency reliability (Cronbach’s α = 0.85). The cutoff scores varied according to years of schooling.

#### PANAS

PANAS is a 20-item two-dimensional scale combining the PA scale, which reflects how an individual feels pleasure or activation, with the NA scale, which describes how a person feels displeased or distressed (Watson et al., 1988). It is scored on a five-point Likert scale. The scales are highly internally consistent and valid and have been widely adopted in studies of aging-related PE (Czerwon et al., 2011; Fairfield et al., 2015).

#### Digit Span

The Digit Span subtest of the Wechsler Adult Intelligence Scale, Fourth Edition (WAIS-IV) (Dumont et al., 2014), which includes two sections, digit-forward and digit-backward, was used to assess working memory ability.

### Stimuli and Task

**FIGURE 1:**
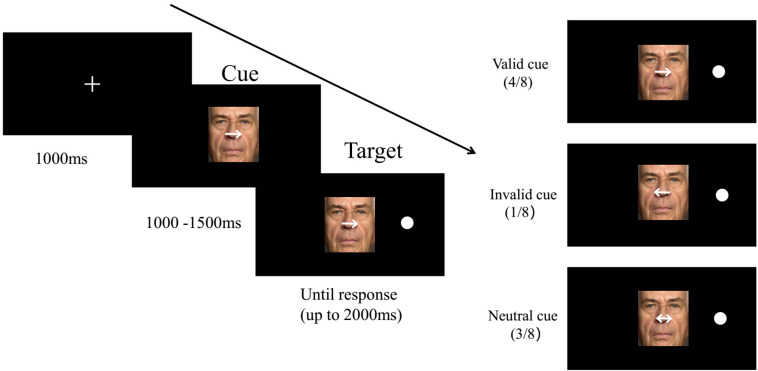
Example trial sequences in the spatial-cueing task.

The stimulus set comprised 96 pictures of 16 older adults (8 men and 8 women) and 16 younger adults (8 men and 8 women) portraying fearful, happy, or neutral facial expressions. All photographs were chosen from FACES, which is a facial expressions database developed by Ebner et al. (2010). To exclude the influence of irrelevant features, all face stimuli were trimmed to 7.0 cm × 10.5 cm using Photoshop CS4 software, with a subtending visual angle of approximately 6.5° × 10°. Hair, ears, neck, and clothes were not included in the images. The modified images contained only the face within a standard rectangular shape (see Figure 1).

The subjects performed the spatial-cueing task modified by Brassen et al. (2011). A trial flow is presented in Figure 1. First, a fixation cross measuring 2 × 2 cm appeared in the middle of the computer screen for 1,000 ms. This was followed by a happy, fearful, or neutral face overlaid with spatially informative or uninformative cues (horizontally 3° of the visual angle) serving as distractors centered in the location of the fixation and randomly presented for 1,000–1,500 ms. Subsequently, a dot target (1.2° × 1.2°) was presented equally often on either the left or right side of the emotional pictures; this dot target was equidistant (horizontally presented at a 5.3° visual angle from the dot target center) from the vertical outer edge of the face and screen, and the face images were always centrally presented. The time window from target onset either terminated when the subjects made a judgment or automatically disappeared at 2,000 ms. The participants were instructed to click their mouse with the left or right index finger to match the location of the target. In one of eight trials, the white arrows were directed to the position opposite that of the target stimulus, which were invalid cues. Consistent with prior studies (Thiel et al., 2004; Brassen et al., 2011), there were very few invalid trials, which were performed only to maintain attention and were excluded from the final analysis. In four of eight trials, the arrows were directed to the ipsilateral side of the target, which were valid cues. The remaining three trials were double-sided arrows, which were neutral cues or uninformative cues, indicating that the probability of the target appearing on the left of the emotional image was equal to that of it appearing on the right. As shown in Figure 1, uninformative cues led to stronger processing of faces and were classified as a high-level attention resource allocated to emotional faces. However, valid endogenous spatial cues may shift implicit attention to the predictive position of the target display, thus reducing attention resources and being categorized as low-level attention to emotional faces.

A total of 32 facial identities (16 male, 16 female; 16 young, 16 old) were selected for this experiment. Each identity (ID) displayed three expressions (fearful, happy, and neutral). The entire experiment took place over four sessions, and each session included 96 trials. Each ID presented all three emotional expressions. A total of 4 × 96 trials were presented for each participant. To avoid vision fatigue, there was a pause or break of 1 min between each session to allow the subjects to rest and shut their eyes. The four sessions were completely counterbalanced.

### Eye Movement Recording

Viewing was binocular, but the movements of the right eye of both old and young participants were gathered using a desktop-mounted Eyelink 1,000 eye tracker (SR Research, Mississauga, Ontario, Canada) at a 1-kHz sampling frequency. The emotional faces were presented on a black background and centered on the screen of a Dell P1130 19-inch monitor (resolution: 1,024 × 768 pixels; refresh rate: 100 Hz). Experiment Builder software (SR Research, Mississauga, Ontario, Canada) was run for the spatial-cueing task presentation. The subjects were tested individually inside a dimly lit, quiet laboratory room and seated 58 cm from the computer screen with a chin and forehead rest. The experiment began with a nine-point calibration, which was repeatedly checked and corrected as needed throughout the experiment.

### Eye-Tracking Analysis and Eye Movement Parameters

**FIGURE 2:**
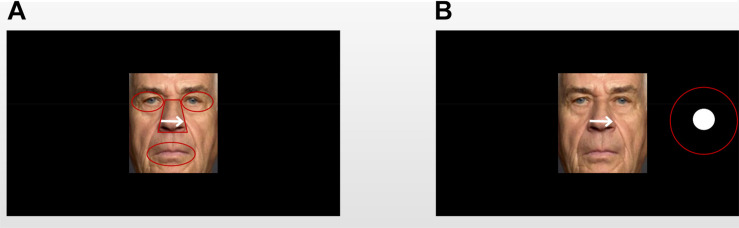
An example of an older adult’s face with the area of interest (AOI) template: **(A)** AOIs of the “cue” faces; **(B)** AOIs of the “target” faces. All AOIs were captured in the same way. In the experiment, the facial expression images and dot targets were displayed in color, and there was no AOI template overlapping the image.

To examine the eye-scanning pattern, the “cue” faces were divided into four areas of interest (AOIs): eyes, nose, and mouth (the salient facial features; Figure 2A). The eyes AOI combined the left and right eyes. Both the eyes and mouth AOIs were portrayed by elliptical shapes that covered each facial feature. The nose AOI was covered by an isosceles-trapezoid area. The size and shape of the whole face AOI were equal to those of the face picture. When the “target” faces appeared, the AOIs were defined as a target stimulus-centered circle with a radius of 1 cm (Figure 2B). The attention engagement stage occurred in the time window in which the “cue” faces were presented, while the attention disengagement stage was measured when the visual target presentation occurred in the time window during which the “target” faces were presented (Hocking et al., 2010; Lester and Vecera, 2018). The indices of the attention engagement stage included three measurements: (1) the proportion of fixation duration and fixations on the AOIs, which was calculated by dividing the fixation duration or fixation on each facial feature (i.e., eyes, nose, and mouth) by the whole picture fixation time or fixation counts during the presentation of “cue” faces, respectively. Attentional disengagement was indexed by (2) saccadic latency, meaning the time window from the onset of the target to the saccade toward the beginning of the target. The criteria for identifying saccade initiation and termination were 22 deg/s velocity and 8,000 deg/s^2^ acceleration, which were embedded in the eye-tracking software (SR Research Ltd.). Saccades below 100 ms or above 1,000 ms were excluded (Leigh and Zee, 1999; Yang et al., 2002).

### Procedure

Prior to the experiment, all participants gave informed consent in conformity with the Institutional Review Board of the Institute of Psychology of the Chinese Academy of Sciences. Their demographic data and neuropsychological functioning were then screened to ensure that they met the inclusion criteria. A series of neuropsychological assessments related to eye-tracking performance (Allard and Isaacowitz, 2008) were performed. The MoCA (Lu et al., 2011) was included as a brief screening instrument for early dementia. PANAS (Huang et al., 2003) was used as a measurement of affect in non-clinical samples, and the Digit Span subtest (Wechsler, 1955) was used to assess fluid intelligence. Finally, the eligible participants underwent the eye-tracking experiment. All subjects received $6 as compensation. The current study was authorized by the Ethics Committee of the Institute of Psychology, Chinese Academy of Sciences.

### Statistical Analysis

The data that support the findings of this study are openly available in Figshare at https://figshare.com/s/3c08c26479186498ed8e, reference number [0–6]. Only trials with correct answers were included in the final analysis. The proportion of incorrect response trials did not differ between the two age groups [*M*_younger_ = 1.13 ± 0.64%, *M*_older_ = 1.28 ± 0.97%; *t*(31) = 0.74, *p* > 0.05]. First, the proportion of the fixation duration and fixations and the saccadic latency for each of the AOIs mentioned above were output using Data Viewer software (SR Research Ltd.) to provide average statistics regarding gaze behaviors during the appearance of “cue” and “target” emotional faces. Second, the IBM-SPSS version 22 software package (IBM, Armonk, NY) was used for repeated-measures analyses of variance (ANOVAs). Greenhouse–Geisser corrected *p*-values were reported when necessary. Third, *post hoc t-*tests were conducted by multiple comparisons following Bonferroni correction adjustment. An *a priori* threshold of *p* < 0.05 was established to indicate statistical significance. The error bars of all bar graphs represent standard errors of the mean (SEM) corrected for repeated measures (Masson and Loftus, 2003).

## Results

The results demonstrated aging-associated cognitive declines in working memory span and cognitive ability (Table 1). However, the PANAS scores for older adults were not significantly different from those for younger adults.

**TABLE 1:** Demographic information and neuropsychological measures (*M* ± SD).

	**Young (*n* = 32)**	**Old (*n* = 32)**	***t*/χ ^2^**	***p***
Age	20.47 ± 1.97	69.94 ± 6.24	42.76	<0.001
Male/female	14/18	12/20	0.26	>0.05
Years of education	13.72 ± 1.09	14.47 ± 2.24	3.36	>0.05
MoCA	27.25 ± 1.97	25.78 ± 1.56	3.31	<0.01
PANAS_PA	34.28 ± 7.04	36.12 ± 6.42	–1.09	>0.05
PANAS_NA	19.88 ± 6.76	17.88 ± 5.33	1.32	>0.05
Digit span	17.56 ± 2.63	13.81 ± 3.96	3.87	<0.001

*PANAS_PA, PANAS positive affect subscale; PANAS_NA, PANAS negative affect subscale.*

### Behavioral Results: Reaction Time Analysis

**FIGURE 3:**
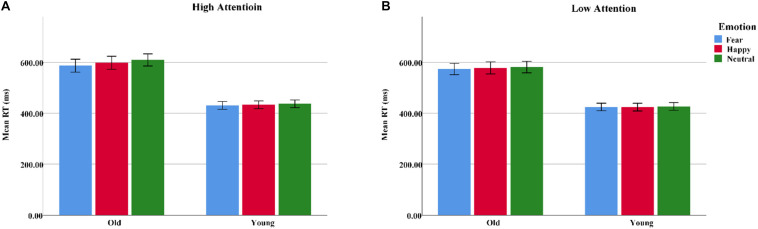
Response times to the target for the spatial-cueing task illustrating emotion (fearful, happy, or neutral) and attention (high, low) in both younger and older adults. **(A)** High attention. **(B)** Low attention. Color blue = Fear; Color red = Happy; Color green = Neutral.

A 2 group (young, old) × 3 emotion (fearful, happy, or neutral) × 2 attention (high, low) ANOVA conducted for response times (RTs) suggested main effects of group [*F*(1, 62) = 31.97, *p*_*correct*_ < 0.001, η^2^ = 0.34], emotion [*F*(2, 124) = 10.38, *p*_*correct*_ < 0.001, η^2^ = 0.14], and attention [*F*(1, 62) = 44.13, *p*_*correct*_ < 0.001, η^2^ = 0.42], as the RTs were generally longer for the older adults than for the younger adults and longer on the low-attention trials than on the high-attention trials. Pairwise comparisons demonstrated a faster RT on fearful trials than on neutral trials [*t*(63) = 4.54, *p* < 0.001]. In addition, the group × emotion interaction was statistically significant [*F*(2, 124) = 3.43, *p*_*correct*_ < 0.05, η^2^ = 0.05] due to the RTs to target, which were significantly shorter on fearful than neutral trials for the older adults [*t*(31) = 4.36, *p* < 0.001]. However, the younger adults showed no emotional differences [*t*(31) = 1.99, *p* > 0.05] (Table 2 and Figure 3).

**TABLE 2:** Mean RTs for emotional faces varying contextual demands among the young and old groups (ms) (means ± SE).

	**Young (*n* = 32)**		**Old (*n* = 32)**	
	**High attention**	**Low attention**	**High attention**	**Low attention**
Fearful	430.66 ± 20.91	424.57 ± 18.92	586.67 ± 20.91	573.45 ± 18.92
Happy	433.11 ± 21.12	423.91 ± 19.56	598.01 ± 21.12	577.92 ± 19.96
Neutral	436.85 ± 19.92	426.67 ± 19.28	609.12 ± 19.92	581.07 ± 19.28

### Gaze Patterns Across Emotions

#### Analysis of “Cue” Faces

**FIGURE 4:**
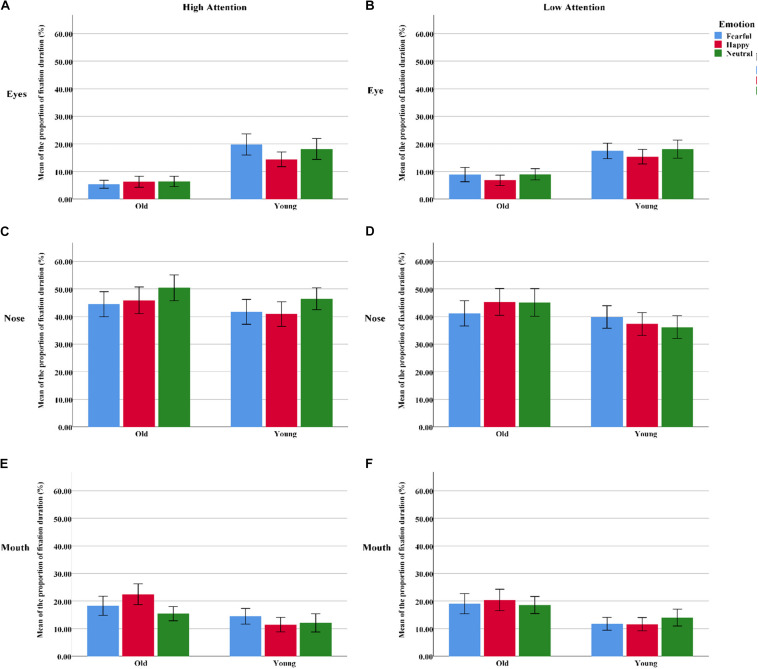
The proportion of fixation duration assigned to the different facial AOIs (eyes, nose, or mouth) for all “cue” faces among the younger and older groups. **(A)** The AOI of eyes under high attention condition. **(B)** The AOI of eyes under low attention condition. **(C)** The AOI of nose under high attention condition. **(D)** The AOI of nose under low attention condition. **(E)** The AOI of mouth under high attention condition. **(F)** The AOI of mouth under low attention condition. Color blue = Fear; Color red = Happy; Color green = Neutral.

The proportion of fixation duration was analyzed with a 2 group (young, old) × 3 emotion (fearful, happy, or neutral) × 3 AOI (eyes, nose, or mouth) × 2 attention (high, low) ANOVA. The results showed a significant AOI effect [*F*(2, 124) = 48.22, *p*_*correct*_ < 0.001, η^2^ = 0.44], as the participants gazed longer at the nose than at the eyes [*t*(63) = 9.12, *p* < 0.001] and mouth [*t*(63) = 6.50, *p* < 0.001] in the “cue” faces. Additionally, significant group × AOI [*F*(2, 124) = 3.60, *p*_*correct*_ < 0.05, η^2^ = 0.06], group × emotion [*F*(2, 124) = 8.22, *p*_*correct*_ < 0.01, η^2^ = 0.12], and AOI × emotion × attention [*F*(4, 248) = 2.44, *p*_*correct*_ = 0.052, η^2^ = 0.04] interactions emerged. Follow-up tests revealed that the proportion of fixation durations within different regions among the older adults was as follows: eyes > nose > mouth (all three pairwise comparisons: *p*s_*correct*_ < 0.004). A similar pattern was found among the younger adults but not for the eyes and mouth regions [*t*(31) = 1.15, *p* < 0.05]. The younger participants gazed longer at fearful faces than at happy faces [*t*(31) = 2.40, *p* > 0.05]. However, the older participants showed opposite fixational patterns [*t*(31) = -2.20, *p* < 0.05]. Under the condition of high attention to faces, no age difference was observed for fearful faces; however, older adults looked at the mouths of happy faces for significantly longer than the younger adults did [*t*(62) = 2.40, *p* > 0.05]. Meanwhile, the nose region received a larger percentage of fixation durations for neutral faces than for happy [*t*(63) = 2.60, *p* < 0.05] and fearful faces [*t*(63) = 2.62, *p* < 0.05]. Nevertheless, there were no emotional effects for the nose region of low-attention trials [*F*(2, 126) = 0.17, *p*_*correct*_ > 0.05, η^2^ = 0.003] (Table 3 and Figure 4).

**TABLE 3:** Summary for the proportion of fixation duration and fixations within each AOI (*M* ± SE).

		**Attention**	**Young (*n* = 32)**		**Old (*n* = 32)**	
			**Proportion of fixation duration (%)**	**Proportion of fixations (%)**	**Proportion of fixation duration (%)**	**Proportion of fixations (%)**
Eyes	Fearful	High	19.85 ± 21.64	9.54 ± 9.11	5.39 ± 8.32	2.56 ± 3.09
		Low	17.49 ± 15.90	9.18 ± 8.13	8.88 ± 14.52	2.83 ± 3.02
	Happy	High	14.42 ± 15.11	9.16 ± 9.14	6.30 ± 11.36	2.05 ± 3.04
		Low	15.39 ± 15.09	8.23 ± 7.62	6.85 ± 10.63	2.19 ± 3.32
	Neutral	High	18.20 ± 21.55	8.36 ± 8.52	6.43 ± 10.36	2.69 ± 3.82
		Low	18.13 ± 18.43	9.78 ± 9.18	9.00 ± 11.40	2.46 ± 3.01
Mouth	Fearful	High	14.49 ± 16.15	6.74 ± 8.09	18.30 ± 19.67	11.20 ± 13.85
		Low	11.81 ± 13.21	6.28 ± 7.85	19.06 ± 20.77	11.47 ± 13.51
	Happy	High	11.46 ± 14.92	6.74 ± 8.17	22.47 ± 21.29	12.84 ± 14.50
		Low	11.62 ± 13.59	6.59 ± 7.60	20.41 ± 21.97	11.59 ± 13.17
	Neutral	High	12.09 ± 18.70	5.51 ± 7.18	15.44 ± 14.56	10.60 ± 11.89
		Low	14.05 ± 17.32	6.03 ± 6.86	18.57 ± 17.49	11.77 ± 13.99
Nose	Fearful	High	41.70 ± 25.45	32.52 ± 22.05	44.46 ± 25.79	37.90 ± 24.13
		Low	39.84 ± 23.17	33.08 ± 21.54	41.19 ± 25.90	34.63 ± 22.83
	Happy	High	40.90 ± 25.25	34.12 ± 22.22	45.90 ± 27.16	37.53 ± 24.74
		Low	37.32 ± 23.19	32.47 ± 21.51	45.28 ± 27.48	36.90 ± 23.45
	Neutral	High	46.45 ± 22.49	37.63 ± 21.12	50.42 ± 26.49	40.33 ± 22.31
		Low	36.15 ± 23.27	32.44 ± 21.65	45.10 ± 26.13	36.48 ± 23.98

**FIGURE 5:**
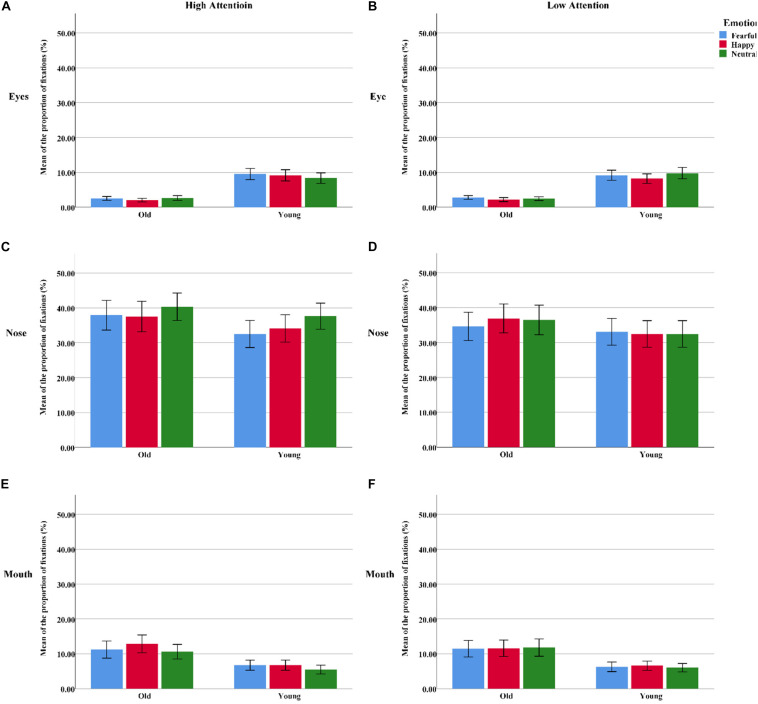
The proportion of fixations assigned to the different facial AOIs (eyes, nose, or mouth) for all “cue” faces among the younger and older groups. **(A)** The AOI of eyes under high attention condition. **(B)** The AOI of eyes under low attention condition. **(C)** The AOI of nose under high attention condition. **(D)** The AOI of nose under low attention condition. **(E)** The AOI of mouth under high attention condition. **(F)** The AOI of mouth under low attention condition. Color blue = Fear; Color red = Happy; Color green = Neutral.

We also performed a repeated-measures 2 group (young, old) × 3 emotion (fearful, happy, or neutral) × 3 AOI (eyes, nose, or mouth) × 2 attention (high, low) ANOVA on the proportion of fixations. The effect of emotion [*F*(2, 124) = 4.69, *p*_*correct*_ < 0.05, η^2^ = 0.07] was more prominent, as a higher proportion of fixations was directed toward neutral faces than toward fearful faces [*t*(63) = 3.35, *p* < 0.01]. The significant main effects of AOI [*F*(2, 124) = 63.42, *p*_*correct*_ < 0.001, η^2^ = 0.51] revealed that the nose was allocated the greatest proportion of fixations, followed by the eyes [*t*(63) = 10.02, *p* < 0.001] and mouth [*t*(63) = 7.21, *p* < 0.001]. The proportions of fixations made to high-attention faces were higher than those to low-attention faces, as supported by a significant attention effect [*F*(1, 62) = 23.75, *p*_*correct*_ < 0.001, η^2^ = 0.28]. Two significant two-way interactions, i.e., emotion and AOI [*F*(4, 124) = 7.50, *p*_*correct*_ < 0.001, η^2^ = 0.11] and AOI and attention [*F*(2, 124) = 20.40, *p*_*correct*_ < 0.001, η^2^ = 0.25]; a significant three-way interaction of emotion, AOI, and attention [*F*(4, 248) = 5.67, *p*_*correct*_ < 0.01, η^2^ = 0.08]; and a significant four-way interaction of emotion, AOI, attention, and age [*F*(4, 248) = 3.51, *p*_*correct*_ < 0.05, η^2^ = 0.05] were demonstrated. Subsequently, six separate ANOVAs were performed for the proportion of fixations assigned to each inner facial AOI of the younger and older participants separately. Both emotion (happy, fearful, or neutral) and attention (high, low) were regarded as within-subject factors for each analysis (Heisz and Ryan, 2011). The findings are shown in Table 3 and Figure 5.

##### Eyes

For the younger adults, the proportion of fixations within the eye region was marginally larger on fearful faces than on neutral faces under high-attention conditions [*t*(31) = 2.05, *p* < 0.05], but this effect was not observed among the older adults [*t*(31) = -0.63, *p* > 0.05]. Meanwhile, the younger adults spent a larger proportion of fixations on neutral faces than on happy faces under low-attention conditions [*t*(31) = 2.37, *p* < 0.05]. However, there were more fixations on fearful faces than on happy faces among the older adults [*t*(31) = 2.05, *p* < 0.05].

##### Nose

The distribution of fixations over the nose area was higher in high-attention trials than in low-attention trials for both fearful [*t*(31) = 3.17, *p* < 0.01] and neutral emotions [*t*(31) = 4.08, *p* < 0.01] for the older participants. This effect was found among the younger adults only in the neutral trials [*t*(31) = 4.46, *p* < 0.001]. Both groups directed a greater proportion of fixations to neutral faces than to fearful [*t*(31) = 4.13, *p* < 0.001; *t*(31) = 2.05, *p* < 0.05] and happy faces [*t*(31) = 2.89, *p* < 0.01; *t*(31) = 2.75, *p* < 0.05] under high-attention conditions.

##### Mouth

Under high-attention conditions, within-group analyses demonstrated that the younger adults allocated a marginally greater proportion of fixations to the mouth for fearful faces than for neutral faces [*t*(31) = 2.03, *p* = 0.051]. Conversely, we found that the proportion of fixations was significantly larger for happy faces than for fearful [*t*(31) = 2.76, *p* < 0.05] and neutral faces [*t*(31) = 2.79, *p* < 0.01] among the older adults. Between-group analyses indicated that the older adults allocated a greater proportion of fixations to the mouth of happy faces than did the younger adults [*t*(62) = 2.07, *p* < 0.05]. However, no such difference emerged for fearful faces [*t*(62) = 1.57, *p* > 0.05].

#### Analysis of “Target” Faces

**FIGURE 6:**
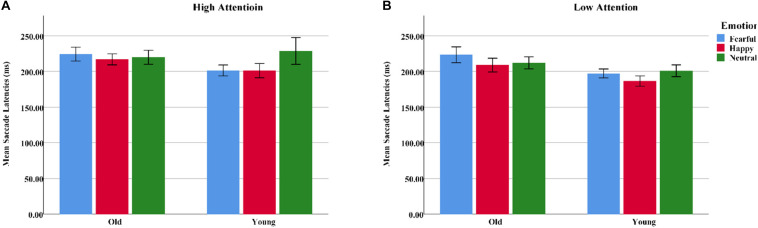
Mean saccadic latencies for emotional faces presented under high- and low-attention conditions among younger and older adults. **(A)** High attention. **(B)** Low attention. Color blue = Fear; Color red = Happy; Color green = Neutral.

Mean saccadic latencies were analyzed with a 2 group (young, old) × 3 emotion (happy, fearful, or neutral) × 2 attention (high, low) ANOVA. Both emotion [*F*(2, 124) = 3.54, *p*_*correct*_ < 0.05, η^2^ = 0.05] and attention [*F*(1, 62) = 5.74, *p*_*correct*_ < 0.05, η^2^ = 0.09] produced main effects. Pairwise comparisons indicated that saccadic latencies were longer on fearful [*t*(31) = 2.34, *p* < 0.05] and neutral faces [*t*(31) = 2.43, *p* < 0.05] than on happy faces, while low-attention trials were significantly shorter than high-attention trials. A significant group × emotion interaction was also observed [*F*(2, 124) = 3.51, *p*_*correct*_ < 0.05, η^2^ = 0.05]. Follow-up tests demonstrated that the younger adults had shorter saccadic latencies on happy faces than on neutral faces [*t*(31) = 2.50, *p* < 0.05]. Notably, fearful faces were associated with longer saccadic latencies than were happy [*t*(31) = 2.50, *p* < 0.05] and neutral faces [*t*(31) = 2.05, *p* < 0.05] for older adults. Independent sample *t*-tests indicated that the older adults had longer saccadic latencies on fearful faces than did the younger adults [*t*(62) = 2.23, *p* < 0.05]. Happy faces, however, showed no age difference [*t*(62) = 1.717, *p* > 0.05] (Table 4 and Figure 6).

**TABLE 4:** Mean saccadic latencies of young and old adults (ms) (*M* ± SE).

	**Attention**	**Saccadic latency**	
		**Young (*n* = 32)**	**Old (*n* = 32)**
Fearful	High	201.33 ± 43.54	224.15 ± 55.20
	Low	197.18 ± 35.19	223.29 ± 62.85
Happy	High	201.10 ± 56.58	216.87 ± 43.35
	Low	186.52 ± 40.88	208.85 ± 54.51
Neutral	High	228.65 ± 105.94	219.83 ± 55.84
	Low	200.89 ± 46.76	211.98 ± 47.87

## Discussion

A number of findings have revealed that positive images draw more attention than negative images for older adults but not for younger adults (Reed et al., 2014). However, whether this age-related PE depends on cognitive control and at which attentional stages it occurs have been unclear. In the current study, we took advantage of eye tracking to investigate the role of cognitive control during implicit attention processing by comparing older adults with younger subjects. We supposed that uninformative (or neutral) cues would allow more available attention resources to engage in stronger face processing, whereas the presentation of highly valid directing cues would direct attention resources toward the cued location and away from the facial images. Thus, we hypothesized that at the attention engagement stage, older adults would be more likely to be distracted by happy faces under the high-attention condition and the attention disengagement stage, and age-related PE would not be observed when attention resources were depleted by covert endogenous cues.

We found that resource-dependent PE was closely related to distinct attentional processes: when more attentional resources were available, the younger adults demonstrated greater attentional deployment to threatening pictures, while the older adults did not display emotional differences at the attention engagement stage. Meanwhile, the older adults fixed longer on the mouth region of happy faces than their younger counterparts. Under low-attention conditions, the older adults even showed a higher proportion of attention engagement to the eye region on fearful faces than on happy faces. Different gaze patterns of bias appeared at the attention engagement stage, which was consistent with the second-generation SST in that age-related PE was dependent on sufficient cognitive control resources. In addition, the hypothesis that the PE of the older adults would not be observed at the attention disengagement stage was confirmed by the saccadic latencies of the older adults, which were longer on fearful trials than on happy and neutral trials at this stage.

### Manual RT Data

The analysis of manual RT data indicated that both the younger and older adults responded faster in the high-attention trials than in the low-attention trials. The spatially informative cues reduced the reaction times, as directional cues may lead to covert stimuli. Under the neutral, bidirectional cue condition, spatial attention was directed to either the left or right side of the display rather than a specific location. This breadth of attentional range enabled the subject to dwell on emotional faces. Based on these findings incorporating analyses of reaction times, we can speculate that more cognitive resources were available to enhance the processing of emotional faces under the undirected cue condition. In addition, our behavioral results suggest that the older subjects showed decreased distractibility by fearful faces under both high-attention and low-attention conditions. In other words, this effect apparently did not depend on cognitive control processes and was inconsistent with the CCH of second-generation SST (Charles and Hong, 2017). A possible explanation is that RT-based measures generally reflect the output stage of manual responses, which occurs between stimulus onset and execution of the response and involves perceptual processing and response selection (Santangelo and Spence, 2008). The spatial-cueing task permits differentiation between attentional engagement and disengagement while processing emotional faces. The low sensitivity of RT-based measurements makes it difficult to discern whether engagement or disengagement of attention is responsible for the observed effects. Bannerman et al. (2010) proposed that saccades had an effect on continuous attentional allocation courses, including attention capture and disengagement, whereas manual responses yielded an effect on the disengagement component over longer periods. They may have distinct mechanisms that act on different processing times, leading to disparate findings for saccades and manual responses. Therefore, we moved on to consider the eye-tracking results to address the specific issues described above, involving the attentional engagement and disengagement of the younger and older adults during emotional face processing.

### Eye-Tracking Data: Attentional Engagement Over Time

Previous studies on facial analysis among healthy people have shown that the regions of the eyes and the mouth are crucial for emotion recognition (Eisenbarth and Alpers, 2011; Noiret et al., 2015; Chaby et al., 2017). In addition, the arrow cues were superimposed on the nose region. Therefore, we performed further analyses of the inner features of emotional faces (i.e., eyes, nose, and mouth). The results revealed that the gaze patterns among the older and younger subjects on emotional faces were similar to some extent. During the presentation of the “cue” images, both age groups exhibited a greater percentage of fixation duration and fixation count distribution on the nose than on the eyes and mouth, since the nose area recorded and transmitted the target-related information. This finding indicates that the eyes and mouth were not uniformly dominant in different types of facial expressions. The eyes seemed to be especially important for recognizing fear, while the mouth was crucial for recognizing pleasure (Murphy and Isaacowitz, 2010; Carlson et al., 2016). For example, fear is usually associated with large eyes, and happiness is closely related to a rise of the mouth (Cangöz et al., 2006). However, the younger adults fixated more frequently and spent longer on the eyes than their older counterparts. A possible explanation is that configural face-encoding processes are influenced by aging to a certain extent; for example, older adults find it more difficult to detect configural changes, but only in the eye region and not in the nose and mouth regions (Chaby et al., 2011; Slessor et al., 2014; Meinhardt-Injac et al., 2015). Our gaze behavior analyses also revealed that under neutral cue conditions, that is, when more attention resources were available for emotional faces, the ratio of fixations was distributed more over the eye area on fearful trials than on neutral trials among the younger participants. By contrast, a higher ratio of fixations was spent on the mouth for happy faces than for fearful and neutral faces among the older participants. This finding is consistent with the opinion that age-related PE occurs in one emotional bias pattern in which young adults present negative bias, whereas older adults present positive bias (Langeslag and van Strien, 2009). Our findings demonstrate that age-related PE does occur when older adults have more available cognitive resources. Interestingly, under valid cue conditions, in other words, when attention resources allocated to the faces were depleted by directional arrows, the younger adults fixated more frequently on the eye region for neutral expressions than for happy expressions, while the older adults fixated more frequently on the eyes for fearful expressions than for happy expressions. In other words, the PE seemed not to exist. The different gaze patterns of the two groups and spatial-cueing effects are consistent with the CCH of second-generation SST at the attention engagement stage. A recent report (Noh and Isaacowitz, 2015) documented eye-tracking applications to explore the role of available cognitive resources in PE. When the experimenters manipulated the contextual demands of emotion processing by adding visual and/or auditory distractions to facial pairs, the aging-related positive gaze preferences tended to decrease or even disappeared under the distracting contexts. Moreover, Kennedy et al. (2020) demonstrated that positive information priority occurred early in visual processing, and this priority was reduced when older participants had a working memory load.

### Eye-Tracking Data: Attentional Disengagement Over Time

The present study also found that saccade latency on fear trials was longer for the older individuals than for the younger adults, which indicated slower disengagement from fearful stimuli among the older adults. As opposed to attentional engagement, age-related PE did not appear in the attentional disengagement stage. One possible way to interpret this finding is that covert attention orientation is not only the process of attention shifting but also the automatic interference inhibition process (Tipper et al., 1998). A recent review concluded that covert orientation is commonly preserved across adulthood, even in later life, under many conditions (Erel and Levy, 2016), and prolonged disengagement from processing a distractor has been demonstrated in normal aging (Cashdollar et al., 2013). We found that the older adults’ ability to disengage attention from threat-related stimuli was worse than that of the younger adults. A generalized defect in shifting attention away from threatening stimuli was observable in the older adults, leading to the disappearance of PE. Another possible explanation was that cognitive resources were at least partly depleted by the target-related response when the older subjects performed the spatial-cueing task, which reflects an unnatural and relatively effortless information processing preference. A systematic meta-analysis comprising 100 empirical studies on the topic of the PE indicated that age-related PE is moderated by cognitive processing (Reed et al., 2014). When participants performed those tasks under encouraging naturalistic and unconstrained circumstances (e.g., viewing images or watching TV), the PE was obvious. However, when participants were instructed to process information conditionally (e.g., under divided attention conditions), the PE was not observed. Second-generation SST proposes that top-down attentional control modulates age differences in the processing of emotional information (Charles and Hong, 2017). Pursuing emotion-related goals demands ample cognitive control and the absence of alternate situation-specific goals. We speculated that shifting spatial attention away from emotional faces may result in a reduction of attention resource allocation to emotional expressions. The spatial-cueing task may activate participants’ information-seeking goals *via* experimental instructions while the “target” faces are present; thus, the older adults had fewer chances to convey their inherent positivity.

## Limitations

Several potential limitations of our study should be acknowledged. First, both the younger and older samples were well educated and quite homogeneous; in future work, it would be better to consider a heterogeneous sample in terms of socioeconomic status, health status, and functioning when examining the preference pattern. Second, although we selected faces from younger and older adults simultaneously to avoid the own-age effect, the emotional faces from the FACES database were Caucasian. Given the evidence of other racial effects in facial recognition (Collova et al., 2017), future research should develop a life-span database containing a wide range of East Asian faces of different age groups. Finally, we recorded the fixation patterns of younger and older adults on fixed emotional distractors selected before the experiment. However, it would be interesting to explore the gaze patterns that would arise if the subjects viewed various distractors closely related to the lives of younger and older adults.

## Conclusion

Despite these limitations, the current study is the first to provide insight into the temporal dynamics of emotional faces during different attentional processing stages in a spatial-cueing task using online eye-tracking techniques. The older subjects showed a resource-dependent PE at the attention engagement stage but failed to exhibit a PE at the attention disengagement stage due to the division of cognitive resources related to experimental constraints on information processing. This finding provides further insights into the cognitive mechanisms of PE in older adults and is consistent with second-generation SST; that is, the positivity effect depends on a relatively sufficient cognitive capacity (Charles and Hong, 2017). Beyond a mechanistic understanding of the stage at which cognitive resources influence the attentional prioritization of positive stimuli, the way in which the attentional resources of older adults are deployed has implications for how emotion is downregulated and even how behavior is adjusted to cope with stress (Livingstone and Isaacowitz, 2017). Finally, the current study may also have functional and potential therapeutic implications. As cognitive ability is closely related to the PE, the application of cognitive training targeting cognitive control ability in older adult interventions may benefit cognitive function and exhibit positive transfer effects in PE, thereby promoting active and healthy aging.

## Data Availability Statement

The datasets and ethical authorization for our study can be retrieved from Figshare under the name “The eye-tracking datasets” (https://figshare.com/s/3c08c26479186498ed8e).

## Ethics Statement

The studies involving human participants were reviewed and approved by Ethics Committee of the Institute of Psychology, Chinese Academy of Sciences. The patients/participants provided their written informed consent to participate in this study. Written informed consent was obtained from the individual(s) for the publication of any potentially identifiable images or data included in this article.

## Author Contributions

HainL contributed to the conception and design of the work, contributed to the writing of the code and implementing parts of the study in E-Prime, and collected the data. HaihL, HainL, and FL contributed to the data analysis and interpretation of the data. HainL and HaihL contributed to the writing of the manuscript draft and revising the work. BH and CW revised the work. All authors contributed to the article and approved the submitted version.

## Conflict of Interest

The authors declare that the research was conducted in the absence of any commercial or financial relationships that could be construed as a potential conflict of interest.

## Footnotes

1 http://jakewestfall.org/power/

2 www.mocatest.org
